# Effect of Jellyfish Body Parts and Presentation Form on Consumers Liking, Sensory Perception, Emotions, and Food Pairings

**DOI:** 10.3390/foods13121872

**Published:** 2024-06-14

**Authors:** Chiara Nervo, Claudia Ragazzini, Luisa Torri

**Affiliations:** University of Gastronomic Sciences, Piazza Vittorio Emanuele II 9, 12042 Pollenzo, Italy; c.nervo@unisg.it (C.N.); claudia.ragazzini@hotmail.com (C.R.)

**Keywords:** alternative proteins, acceptability, drivers of preference, check-all-that-apply question, consumer segmentation, penalty analysis, sustainable food source

## Abstract

Although jellyfish represent a food source in Asia, limited attention has been devoted to investigating Western consumers’ perception and acceptance. This study explored the role of jellyfish body parts and presentation form in determining consumer perception. A local consumer test with 106 untrained subjects (57.5% female, 18–45 years) was performed in Italy over two days on six samples of jellyfish (*Rhopilema esculentum* Kishinouye) differing in terms of body parts (umbrella and oral arms) and presentation form (minced, striped, and pieced). For each sample, participants expressed their overall liking and, through three check-all-that-apply tests, described their perceived sensory properties and emotions and potential preferred food pairings. The results showed a significant effect of presentation form on liking (with striped and minced samples liked more than pieced samples), 18 sensory properties, four emotions, and five food pairings. Moreover, different drivers of liking and emotions were observed for three clusters of subjects named “In favour of”, “Against”, and “Picky towards” eating jellyfish. In conclusion, this study found that at least one segment of consumers could accept jellyfish as novel food. Moreover, the provided results could be useful for developing innovative jellyfish-based products and dishes that meet consumers’ expectations.

## 1. Introduction

Jellyfish have been consumed as food in China and other Asian countries (e.g., Japan, India, Philippines, and Malaysia) for centuries [1]. The most consumed jellyfish species in Asia (*Rhopilema esculentum*, *Rhopilema hispidum*, and *Nemopilema nomurai*) are part of the Rhizostomatidae family, including the genera Rhopilema, Nemopilema, and Rhizostoma [2]. The main anatomical parts of jellyfish are the umbrella (body), the oral arms (legs), and the tentacles. The first two are the most relevant edible jellyfish body parts [3], with oral arms being richer in proteins and lipids than the jellyfish umbrella [4]. Jellyfish are mainly consumed after being treated, although some species are also eaten as fresh ingredients in China, including *Rhopilema esculentum* and *Nemopilema nomurai* [5]. After arriving at a processing facility, the umbrella and the oral arms are processed separately [6]; the former is the most requested part and, therefore, also the most expensive, but requests for oral arms have increased in recent years [7]. After rinsing them and removing mucus, the gastrovascular cavity, and developed gonads, jellyfish are left in contact with mixtures of different concentrations of alum and salt for about 20–40 days [8]. This long and inexpensive treatment allows for dehydrating and disinfecting the jellyfish, thus obtaining a finished product with a long shelf life (about 1 year at room temperature), characterized by a particular texture loved by Asian populations, which does not need to be cooked to be consumed [9]. They are sold on the market both in brine and ready-to-eat [10]. Before consumption, jellyfish sold in brine must be desalted for a few hours in fresh water. This product is appreciated firstly for its peculiar texture and secondly for its taste [9]. Traditionally, its rigid and crunchy texture is enhanced by preparing the jellyfish mainly by cutting them into thin strips and pairing them with raw vegetables in a salad seasoned with sesame oil, sugar, soy sauce, and rice vinegar [3].

Although jellyfish consumption is traditional in Asian countries, jellyfish represent an unfamiliar source of food for Western consumers in general, and especially for European consumers, since jellyfish are not available in EU countries because of their status as “novel food”, thus requiring EU authorization before being placed on the market [11]. While research on jellyfish as food has mainly focused on nutritional properties, safety issues, processing technologies, and potential applications [12,13,14,15], limited attention has been devoted to the investigation of consumers’ sensory perception and acceptance of jellyfish or jellyfish-based products [15].

In the recent past, a panel of Italian trained assessors developed a sensory lexicon specific for edible jellyfish and used it in a descriptive analysis to define the sensory profile of *Rhizostoma Pulmo* sampled that underwent different mild treatments [16]. A descriptive analysis was also applied by a Portuguese panel of trained assessors to evaluate the sensory profile of four pâté samples prepared by mixing small pieces of the *Catostylus tagi* jellyfish umbrella with different proportions of mayonnaise [3]. In the same study, a questionnaire was administered to Portuguese students to assess their interest in jellyfish, the type of dish preferred, and the motivations behind it. Recently, research was carried out to compare the likelihood of adopting alternative proteins, including jellyfish, insects, and cultured meat, by Canadian consumers [17]. Similarly, the variables influencing the attitude towards jellyfish of Italian consumers were compared with those impacting the acceptance of other novel foods such as insects and seaweeds [18]. In particular, they explored the attitude towards jellyfish taking into account different gastronomic modalities of consumption, including forms of consumption, food preparations, use as ingredients in processed foods, and more suitable food pairings with other foods. With a similar gastronomic approach, another study explored the role of individual variables such as socio-demographic characteristics (gender, age, nationality, educational level, geographic area of residence, and the number of international trips per year), food habits, and personality traits (food neophobia, disgust sensitiveness) on the consumers’ attitudes towards jellyfish as potential food in Italy considering the visible appearance of the jellyfish body, the ingredient role of jellyfish in the dish, the carrier flavour of the recipe in which jellyfish could be used, and potential food pairings [19]. The same methodological approach was recently applied to cross-culturally compare the potential of jellyfish as a sustainable food source in nine Latin American countries [20]. The last three cited studies, using an innovative methodological approach centred on the gastronomic perspective, provided useful insights for the future development of jellyfish products by increasing the probability of meeting the expectations of consumers interested in alternative protein food sources. However, in the last study, the authors recommended that forthcoming works comprise sensory tests to better understand consumers’ sensory and hedonic perceptions of jellyfish.

Based on the above, it was hypothesized that jellyfish body parts and the presentation form could influence consumers’ affective responses. Therefore, this research aimed to explore the role of the jellyfish body parts (umbrella vs. oral arms) and presentation form (pieced, striped, minced) in determining consumers’ responses in terms of liking, sensory descriptions, emotions, and preferred food pairings.

## 2. Materials and Methods

### 2.1. Jellyfish

*Rhopilema esculentum* Kishinouye (*R. esculentum*) was selected for this study since, among the several species of Scyphozoan jellyfish, it is the most abundant and most important edible jellyfish species in the Asian jellyfish fishery and market [2].

In particular, commercial salted jellyfish oral arms and jellyfish umbrellas were selected. Based on what is reported on their labels, both commercial products were fished in the Pacific Ocean using trawls, immediately treated with salt and alum, and preserved in a brine of water and salt. The samples were sold packaged in large plastic jars with lids (net weight: 1850 g; drained weight: 800 g). In the original containers, the arms were stored as cut pieces about 8 cm long, and the umbrellas were into strips of about 6 cm. The products were marketed by the company Ykof (Yingkou City, Liaoning, China) and imported to Italy by the Dingfeng ImportExport S.r.l. (Novate Milanese, Italy).

Before being administered to consumers for the sensory evaluation, the oral arms and umbrella parts were desalted separately for two hours in plastic bowls filled with fresh water. To eliminate most excess salt, the water was changed three times every 30 min, and then the jellyfish oral arms and umbrellas were drained.

**Figure 1:**
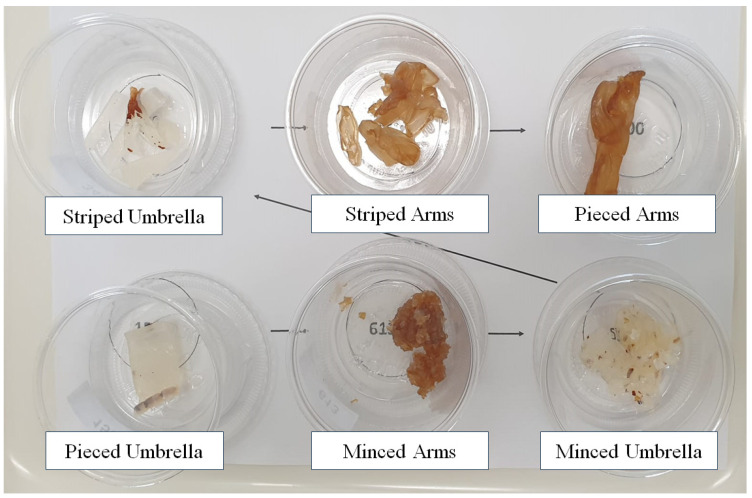
Image of the six samples of jellyfish differing in terms of body parts (oral arms and umbrellas) and presentation form (pieced, striped, minced).

The oral arms and the umbrella products were presented in three different forms (pieced, striped, and minced) to verify if appearance plays a key role in jellyfish acceptability. Thus, six samples were tested as follows: pieced umbrella, pieced arms, striped umbrella, striped arms, minced umbrella, and minced arms (Figure 1). The pieced umbrella and pieced arm samples were obtained simply by cutting the commercial products with a knife into pieces of about 2 cm. The striped umbrellas and striped arms were obtained by cutting the products with a knife into thin strips about 0.5 cm thick, while the minced umbrella and minced arm samples were processed with a blender into very small pieces of 2–3 mm, similar to a tartare.

The samples were served in their natural form, without any seasoning, to collect information on the sensory perception of the plain product without the influences of any other ingredients.

The jellyfish samples were presented in 96 mL plastic containers covered with a plastic lid, identified by three-random digit codes, and in a randomized and balanced order among subjects.

### 2.2. Participants

Eight subjects (5 females and 3 males, mean age: 21.6 ± 2.3 years, range age: 20–27 years, 100% Italian) participated in a focus group to identify the attributes to use in the consumer test to describe the appearance, aroma, taste, flavour, and texture of jellyfish samples. One hundred and six subjects (Table 1) participated in the consumer test.

All subjects were untrained (thus representative of consumers) and recruited via e-mail among students and staff of the University of Gastronomic Sciences. The researchers gave the participants verbal information prior to conducting the test on the samples. In particular, the participants were informed about the aim of this study and that the samples were jellyfish (commercial products). Moreover, the participants were informed of the procedure to adopt for the evaluation of the samples. The participants were also informed of the following: all information was collected in anonymous form; participation in this research project did not come at any cost; and products that were not harmful to the participants were used in the sensory tests. The participants were required to disclose any specific intolerances, allergies, or aversions before starting the test in order to avoid any possible risks due to exposure. Once the participants decided to join this study, they were informed of the following: they would contribute to a research project conducted by the University of Gastronomic Sciences; there were no disadvantages linked to participating in this study; and this study did not foresee any risks to the participants. Participating in this study was fully voluntary; thus, the participants were advised that they were free to withdraw and interrupt this study at any time without consequences and that any collected information was strictly confidential and protected by professional secret (the release of such information depended on their authorization). The participants voluntarily took part in this study and provided their free and informed consent before beginning the test (Supplementary Material S1). This study was approved by the Ethics Committee of the University of Gastronomic Sciences (Ethics Committee Proceeding n. 2022.01) and carried out following the international ethical guidelines for research involving humans established in the Declaration of Helsinki.

### 2.3. Consumer Test

The consumer test comprised a liking test, three check-all-that-apply (CATA) tests, and a final questionnaire on socio-demographic information [21].

In the liking test, each participant was asked to observe, smell, and taste the samples and then rate their overall liking of each sample using a nine-point hedonic scale ranging from 1 = extremely disliking to 9 = extremely liking [22].

During the first CATA test, the participants were asked to select all sensory attributes perceived for each sample from a list of 40 terms (Table 2) determined during a previous focus group (amber, bitter, cartilaginous, compact/dense, crunchy, dry, elastic, fibrous, fish, fresh/cool, gelatinous, grainy, green vegetables, hard, iron, marine/brackish, mineral, opaque/haze, pet food, porous, salty, seaweed, shellfish, slimy, small dots, smooth, soft, sour, stagnant water, sulphur, sweet, toasted, transparent, umami, uniform colour, vinegar, viscous, watery, white, and wrinkled/rough).

During the second CATA test, the participants were asked to select all food pairings considered suitable for each sample of jellyfish, choosing among 21 items (bakery products, cheese, cooked vegetables, cream, dessert, eggs, fish, fruit, honey/jam, meat, pasta, potatoes, pulses, raw vegetables, rice/risotto, salty snacks, sauces/condiments, spices/aromatic herbs, yogurt, none, I would not eat it, and none, I would eat it alone) previously used [21].

During the third CATA test, subjects were asked to select all the emotions induced by tasting choosing from a list of 39 attributes (active, affectionate, adventurous, aggressive, bored, calm, daring, disgusted, angry, energetic, enthusiastic, free, friendly, glad, good, good-natured, guilty, happy, interested, joyful, loving, merry, mild, nostalgic, peaceful, pleased, pleasant, polite, quiet, satisfied, secure, steady, tame, tender, understanding, warm, whole, wild, and worried) [19,23].

Between samples, the subjects were required to rinse their mouths with still water for one minute to restore their sensory ability.

The final questionnaire aimed to collect information regarding socio-demographic characteristics (gender, age, and nationality), food habits (vegan, vegetarian, omnivore, or flexitarian), and previous tasting of jellyfish.

The consumer test took place in individual sensory booths, under white light, with no social interaction permitted, and lasted about 40 min. Data were collected over two days between 11 am and 4 pm using Fizz software version 47B (Biosystèmes, Couternon, France).

### 2.4. Data Analysis

The liking data from all subjects were analysed with both principal component analysis (PCA) and a two-way ANOVA model (the fixed factors included body part, presentation form, interaction body part*presentation form). Moreover, the liking data were submitted to hierarchical cluster analysis to identify clusters of subjects with similar preferences. A chi-square test was used to verify any differences in terms of subject characteristics among the identified clusters. In addition, a two-way ANOVA was performed to investigate the effect of both the cluster and jellyfish sample on liking (the fixed factors included clusters of consumers and samples). All ANOVA models were followed by Tukey’s HSD test.

To identify differences in terms of sensory attributes, emotions, and possible food pairings of the different samples of jellyfish, the data obtained from the three CATA tests were separately analysed with Cochran’s Q tests, followed by Sheskin’s comparison test for multiple pairs. Moreover, the three occurrence matrices obtained from the three CATA tests were also analysed by correspondence analysis.

Penalty analysis was then conducted on the liking data and the occurrence matrix of the sensory attributes identified as significant in the multiple pairwise comparison tests to assess which sensory attributes positively or negatively affected the liking of the jellyfish samples. A penalty analysis was applied firstly to the data provided by all the subjects and then separately to the data related to the identified clusters. A penalty analysis was also applied to the liking data and the occurrence matrix of the emotions selected by all the subjects to assess which emotions positively or negatively affected the liking of the jellyfish samples. For the emotion data, a chi-square test followed by Fisher’s exact probability test was used to determine if the identified clusters differed in the association between emotions and jellyfish consumption.

All statistical tests were performed at a significance level of 5% with the XLSTAT statistical software package, version 2021.4.1 (Addinsoft, Paris, France).

## 3. Results

### 3.1. Characteristics of the Participants

The socio-demographic characteristics of the 106 subjects involved in this study are listed in Table 1. The majority of the population was female (57.5%), had an age ranging between 18 and 30 years (91.5%), and was Italian (78.3%). Those who were not Italian had different nationalities from all over the world.

More than half of the participants were omnivorous (64.2%), while 34.9% declared that they followed a flexitarian diet. Only one subject was vegetarian (1.6%), while no one declared that they followed a vegan diet. The majority of subjects had never previously tasted jellyfish (84%).

### 3.2. Consumer Acceptance of Jellyfish as Food

**Figure 2:**
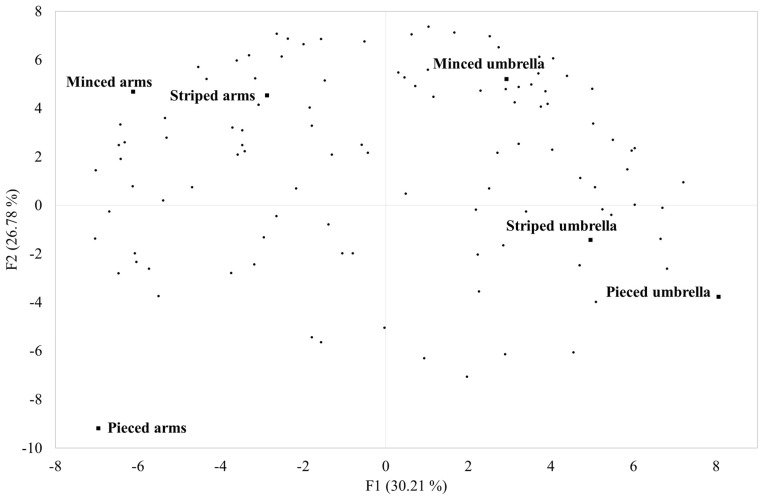
Biplot obtained from the principal components analysis applied to the liking data provided by 106 subjects for six samples of jellyfish differing in terms of parts (arms vs. umbrella) and presentation forms (pieced, striped, minced).

The results from the PCA applied to the overall liking data are depicted in Figure 2. The total variance explained by the biplot was 56.99%, with PC1 and PC2 accounting for 30.21% and 26.78%, respectively. It can be noticed that PC1 discriminated the samples according to the jellyfish parts, with umbrella samples positively correlated to PC1 and arm samples negatively correlated to it. Moreover, the samples were positioned along PC2 according to the form of presentation, from negative values to positive values in the following order: pieced, striped, and minced. The subjects were distributed on the overall surface of the biplot, with most of them positively correlated to PC2, indicating a tendency to prefer the minced umbrella, striped arm, and minced arm samples.

Considering all the subjects, the results of the two-way ANOVA applied to the liking data showed a significant effect (*p* < 0.0001) of both the presentation form factor and the body part*presentation form interaction (Table 3), while no significant effect of the body part factor was found (*p* = 0.689). In particular, the striped and minced presentation forms obtained a significantly higher mean liking value than the pieced presentation form. The striped umbrella, striped arm, minced umbrella, and minced arm samples received a higher mean liking value than the pieced arm and pieced umbrella samples.

The results of the cluster analysis applied to the liking data identified three clusters of subjects as follows: Cluster 1, composed of 40 subjects (37.8%); Cluster 2, composed of 44 subjects (41.5%); and Cluster 3, composed of 22 subjects (20.7%). Although the chi-square test did not reveal any significant differences (*p* > 0.05) among the characteristics of the three clusters (Table 1), significant differences were observed in terms of liking based on the results of the two-way ANOVA models (Table 3). The jellyfish part was not a significant factor for Cluster 1, while it was significant for the other two clusters, with an opposite effect as follows: Cluster 2 preferred the arms over the umbrella, while for Cluster 3, the average value obtained for the umbrella was much higher than that for the arms. Moreover, Clusters 1 and 3 liked the umbrella significantly more than Cluster 2, while Cluster 1 liked the arms more than the other two clusters. Concerning the presentation form, similar to what was observed for all the subjects, for all three clusters, the striped and minced presentation forms obtained a significantly higher mean liking value than the pieced presentation form. Overall, Cluster 1 provided a higher mean value than Clusters 2 and 3. No significant differences were noticed between Clusters 2 and 3 in the pieced presentation form. Oppositely, Cluster 3 obtained a higher liking for both the striped and minced forms than Cluster 2.

### 3.3. Consumer Perception of the Sensory Characteristics of Jellyfish

The sensory attributes most frequently used to describe the samples were appearance attributes, such as amber, opaque/haze, transparent, uniform colour, and white (Table 4). For taste, the most frequently mentioned attributes were salty and umami, while fish, fresh/cool, marine/brackish, mineral, seaweed, shellfish, and stagnant water were the flavour attributes that were mainly mentioned. Finally, the attributes most commonly used to describe the sample texture were cartilaginous, compact/dense, crunchy, gelatinous, hard, and watery.

The Cochran’s Q test applied to the CATA data obtained from the subjects revealed 18 significant attributes. In terms of appearance, the arm samples were significantly more associated with amber, while the umbrella samples were more frequently associated with white. Moreover, the presentation form influenced the perception of a grainy appearance, which was higher in the minced umbrella and arm samples than in the striped and pieced samples. Regarding flavour, shellfish was selected significantly more often for the striped arm sample than for the pieced and minced umbrella samples, while the cartilaginous attribute was selected significantly more often for the pieced umbrella sample than for the minced arm sample. Concerning texture, the crunchy attribute was significantly more associated with the umbrella-based samples than with the corresponding arm-based samples for both the striped and minced presentation forms. Finally, the highest association with hardness was observed for the pieced umbrella sample, while the lowest association was observed for the minced arm sample.

**Figure 3:**
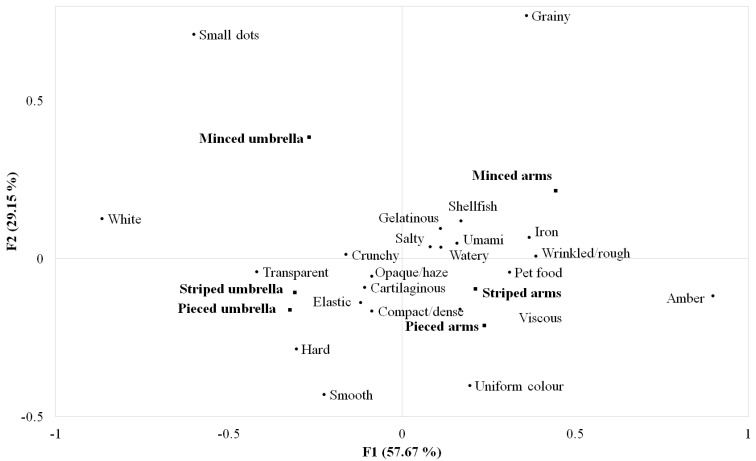
Correspondence analysis ordination diagram showing the associations among the significant sensory attributes and six jellyfish samples differing in terms of parts (arms, umbrella) and presentation form (pieced, striped, minced).

The correspondence analysis ordination diagram summarizing the relationships between the different significant attributes and the six jellyfish samples is shown in Figure 3. The total inertia explained by the CA plot of two-dimensional coordinates was 86.82%, with Factor 1 and Factor 2 accounting, respectively, for 57.67% and 29.15%. Similar to what was previously observed in terms of liking (Figure 2), the samples were discriminated along the first factor according to the jellyfish part, with the arm samples positively correlated with PC1 (thus, mainly described by an amber colour, a wrinkled/rough appearance, and an iron and pet food flavour) and the umbrella samples negatively correlated with it (highly associated to a white and transparent colour). Along with the second factor, the samples were distributed according to the form of presentation, with the minced samples opposite to the striped and pieced samples. From this map, it is evident that the striped umbrella and pieced umbrella samples have an overall comparable sensory profile. The first couple of samples were mainly distinguished by hard and smooth attributes, while the minced umbrella sample was mainly characterized by the presence of small dots on the surface. Likewise, the pieced and striped arms were overall perceived as similar to each other and quite different from the minced arms. While the first two samples were more associated with the uniform colour, viscous, compact/dense, and elastic attributes, the minced arms were more associated with an umami taste, shellfish flavour, and gelatinous texture.

Summarizing the relationships among the sensory variables, the white and amber colour attributes, as well as a uniform colour and small dot appearance, were negatively correlated. Regarding texture, the grainy and smooth attributes were negatively correlated.

#### Impact of Sensory Characteristics on Liking of Jellyfish

**Figure 4:**
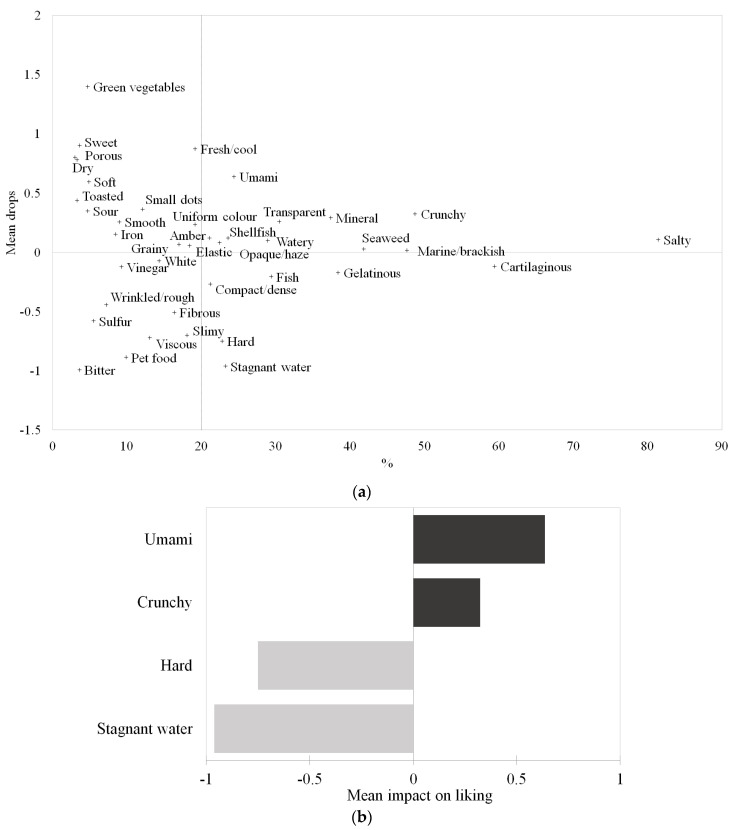
Results of the penalty analysis applied to the liking data and the occurrences of the sensory attributes from the check-all-that-apply-test: (**a**) mean impact of the sensory attributes used to describe the six jellyfish samples and (**b**) sensory attributes with a significant mean impact on liking (mean increases are displayed in dark grey, while mean decreases are displayed in light grey).

The results of the penalty analysis applied to the liking data and the occurrences of the sensory attributes obtained from the check-all-that-apply-test are shown in Figure 4. As reported in Figure 4a, the umami, amber, elastic, shellfish, opaque/haze, watery, transparent, mineral, seaweed, crunchy, marine/brackish, and salty attributes tended to increase the liking of jellyfish. On the contrary, the cartilaginous, gelatinous, fish, compact/dense, hard, and stagnant water attributes seemed to reduce the liking.

As depicted in Figure 4b, the umami and crunchy attributes have a significantly positive average liking impact of 0.3 and 0.6 points, respectively. At the same time, the hard and stagnant water attributes contribute to lowering the mean values of the overall liking of the samples of 0.7 and increasing points.

**Figure 5:**
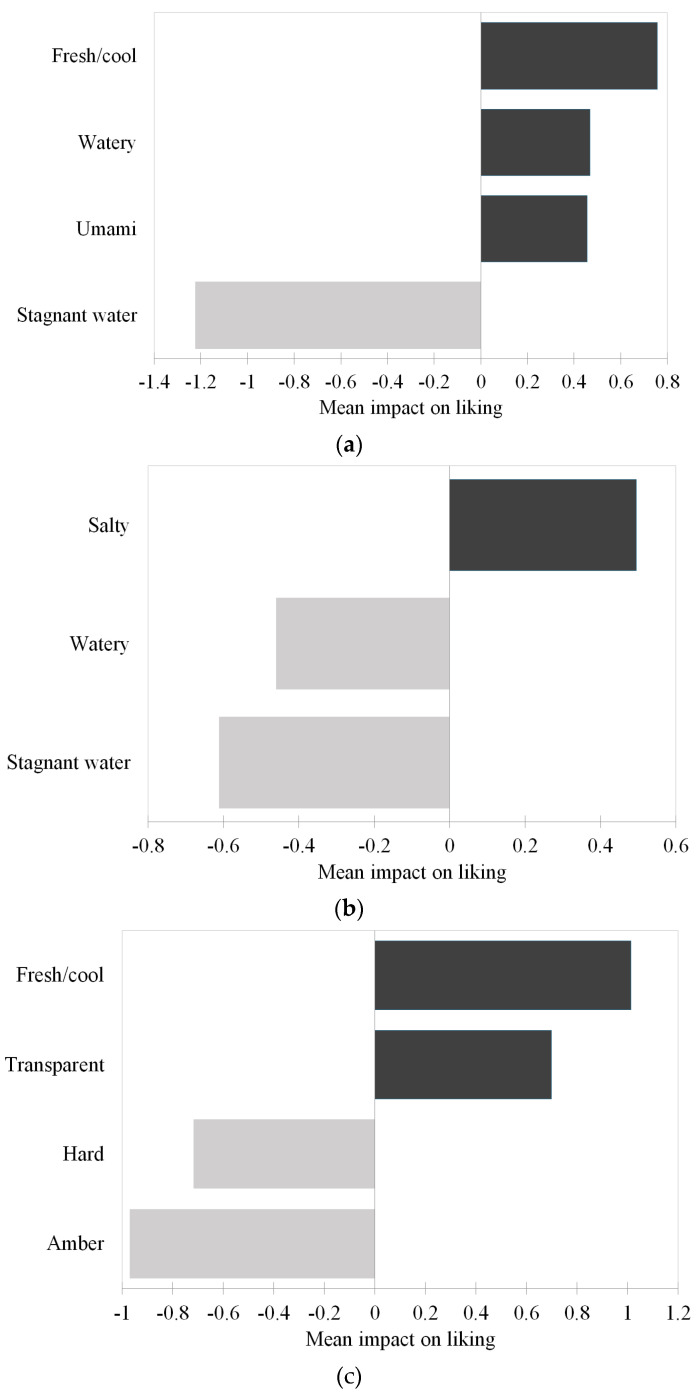
Results of the penalty analysis applied to the liking data and the occurrences of the sensory attributes from the check-all-that-apply test of the three clusters showing the sensory attributes with a significant mean impact on liking. Mean increases are displayed in dark grey, while mean decreases are displayed in light grey. (**a**) Cluster 1; (**b**) Cluster 2; and (**c**) Cluster 3.

When comparing the results of the penalty analysis separately conducted on the three clusters (Figure 5), similarities and differences emerged. Specifically, it was observed that the liking drivers of Cluster 1 (Figure 5a) were fresh/cool, watery, and umami; only the attribute stagnant water was a negative driver, which, if perceived, contributed to decreasing the mean liking by 1.2 points. The latter was also a negative driver of liking for Cluster 2 (Figure 5b). Interestingly, watery, which was a positive driver for Cluster 1, was found to be a negative driver for Cluster 2. The other positive driver for Cluster 2 was salty, which increased the mean liking value by 0.5 points. Lastly, the attributes that positively affected the mean liking of Cluster 3 were transparent and fresh/cool (also relevant for Cluster 1), while the negative drivers were hard and amber, which contributed to a decrease in the mean value of 0.7 and 1.0 points, respectively (Figure 5b).

### 3.4. Emotions Associated with Jellyfish Consumption

The emotions mostly associated with jellyfish, regardless of the body part and presentation form, were adventurous, calm, disgusted, enthusiastic, interested, satisfied, wild, and worried (Table 5). The Cochran’s Q test applied to the CATA data obtained from the subjects revealed four significant attributes as follows: (1) free, with a higher number of occurrences for the minced umbrella sample compared with the pieced umbrella sample; (2) glad, significantly more associated with the minced arm sample than for the pieced umbrella sample; (3) satisfied, more associated with the striped umbrella sample than pieced arms; and (4) worried, mostly associated with both pieced samples.

The results of the penalty analysis applied to the liking data and the occurrences of the emotions obtained from the check-all-that-apply-test by all the subjects revealed only the following two emotions with significant impact on the liking of the jellyfish samples: interested, which raised the mean liking by 1.0 points, and disgusted, which lowered it by 2.1 points.

The results of the chi-squared test and Fisher’s exact probability test allowed for the identification of significant differences among the three identified clusters (χ2 = 202.6; *p* < 0.0001). As shown in Table 6, 13 out of the 39 emotions were significant in discriminating among clusters. In particular, for Cluster 1, a significantly higher proportion of enthusiastic, free, happy, pleasant, and satisfied emotions, and a lower proportion of adventurous, disgusted, quiet, and worried emotions, were observed. Contrarily, Cluster 2 was significantly more associated with adventurous, disgusted, wild, and worried emotions and less associated with energetic, enthusiastic, happy, pleasant, satisfied, and secure emotions. Lastly, for Cluster 3, the only significantly higher proportion observed was for the affectionate emotion.

### 3.5. Most Appropriate Pairing with Jellyfish

Observing the total occurrences of potential food pairings for jellyfish (Table 7), it is possible to notice that the most frequently chosen attributes, without distinction of body part and presentation form, were cooked vegetables, fish, pasta, potatoes, raw vegetables, rice/risotto, sauces/condiments, savoury snacks, and spices/aromatic herbs.

The Cochran’s Q test applied to CATA data obtained from the subjects revealed five significant attributes. In particular, pasta was selected significantly more often for the minced arm sample than for the pieced umbrella and arm samples. Similarly, potatoes were paired significantly more often with the minced arm sample than with the pieced umbrella sample, while rice/risotto was especially associated with both minced samples. The response “None, I would not eat it” was selected significantly more often for the pieced samples, suggesting that consumers probably would not be willing to eat this jellyfish sample again.

**Figure 6:**
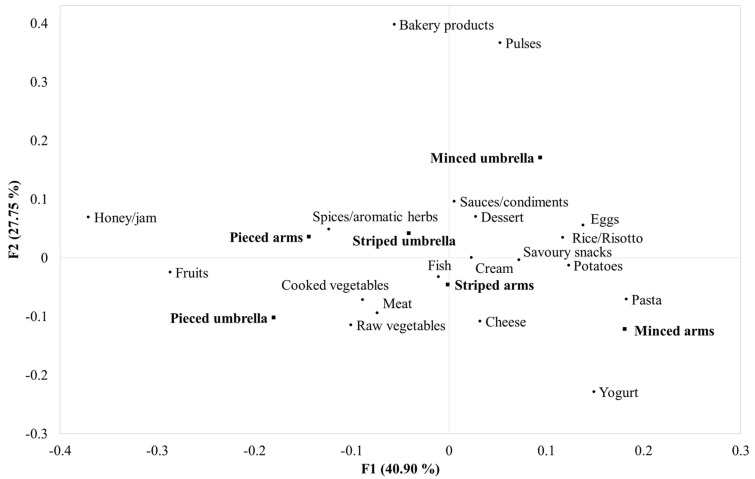
Correspondence analysis ordination diagram showing the association among possible food pairings and the six jellyfish samples differing in parts (umbrella, arms) and presentation form (pieced, striped, minced).

The correspondence analysis ordination diagram that shows the relationships among the different food pairings and the six jellyfish samples is reported in Figure 6. For this statistical analysis, the options “None, I would not eat it” and “None, I would eat it alone” were not included. The total inertia explained by the CA plot of two-dimensional coordinates was 68.65%, with Factor 1 and Factor 2 accounting, respectively, for 40.90% and 27.75%. This map showed that the samples were distributed along Factor 1 based on the presentation form, with the order minced, striped, and pieced from positive values to negative values of the first factor. The minced arm sample was mostly associated with yogurt, pasta, cheese, and potatoes, while the minced umbrella sample was mostly paired with sauces/condiments and dessert. The striped umbrella and striped arm samples were possibly paired with similar foods, especially with spices/aromatic herbs, fish, cooked vegetables, meat, and raw vegetables. Also, the pieced arm and pieced umbrella samples had comparable food pairing options, with higher preferences for honey/jam and fruits.

## 4. Discussion

### 4.1. Hedonic Response towards Jellyfish as Food

The present study represents the first (to the best authors’ knowledge) to report results regarding the sensory perception of and affective response to jellyfish as a food source by Italian consumers. We note that a sensory work was recently conducted in Italy on samples of *Rhizostoma pulmo* jellyfish that underwent different mild treatments, with the threefold aim to develop a sensory lexicon for edible jellyfish, define the sensory profiles of the treated samples, and investigate their liking [16]. However, that study did not involve consumers but a panel of nine trained assessors. Even if some hedonic data were collected, the application of the liking test with nine trained subjects seems unusual in the sensory science field (since it does not represent the consumer response), as it is recommended to conduct hedonic tests with a high number (at least 50) of untrained consumers [24].

The liking obtained from all the subjects for the six samples of *R. esculentum*, which differed in terms of body parts (umbrella vs. oral arms) and presentation form (pieced, striped, minced), was on average quite low and did not reach acceptability (value equal to five on the used nine-point hedonic scale). This result could be partially explained by two possible reasons. Firstly, it is relevant to consider that most participants were unfamiliar with jellyfish since 84% of them had never tasted jellyfish before. Some authors [25] proposed that exposure is the main building block of food familiarity, and it is known that familiarity plays a crucial role in promoting the acceptance of novel foods since consumers are programmed from an early age to prefer familiar foods [26]. The proportion of subjects in the present study who previously tasted jellyfish (16%) was higher than reported in the literature. Indeed, in the studies investigating the attitude towards jellyfish as potential food through online surveys, 5% of Italian subjects [19] and 3.8% of Latin Americans (with values ranging from 0.4% for El Salvador to 8.1 for Mexico [20]) declared having already eaten jellyfish. An even lower proportion (1.6%) was observed among respondents participating in research conducted in Portugal [3]. Secondly, jellyfish was evaluated as a plain product, allowing for the description of the natural sensory properties and avoiding any possible influences of any seasoning or other added ingredients. It could be assumed that liking would increase if the jellyfish samples were tasted as a seasoned version (e.g., with oil and vinegar) to simulate a more ecological consumption context. However, the obtained liking results are more positive than those reported for three marinated semi-dried jellyfish products (from the *Catostylus mosaicus* species) seasoned with three options (sesame, sesame and ginger, soy sauce), which received average overall liking scores ranging from 15 to 28 on a 100-point scale (0 = dislike extremely, 50 = neither like nor dislike, 100 = like extremely) by Asian consumers [27]. It could be argued that jellyfish used as an ingredient in a food product could improve consumer acceptance. This was the case for the pâté snacks made using different concentrations (5–25%) of small pieces of jellyfish umbrella with mayonnaise, which were accepted by 90% of consumers with a mean liking of 6 (like moderately) on the nine-point hedonic scale [3]. Similarly, nuggets prepared with 10, 20, and 30% jellyfish replacing catfish meat obtained an average liking of 3.5–3.8 on a five-point hedonic scale, corresponding to an evaluation between neutral (3) and like (4) [28]. Finally, crackers made by replacing wheat flour content with ground jellyfish (20–40% *w*/*w* of total wheat) reached a mean liking score of 6.1–6.4 on a seven-point hedonic (1 = extremely dislike, 7 = extremely like) [29].

The results of the cluster analysis revealed several overall liking differences, with Cluster 1 accepting all jellyfish samples independently of the body part and presentation form. Contrarily, Cluster 2 did not accept jellyfish samples at all, and Cluster 3 discriminated the most among samples, accepting the minced umbrella sample but disliking very much the pieced arm sample and slightly disliking the other jellyfish samples. A very similar segmentation result was observed in a previous study [30] conducted with Italian consumers on another category of novel foods, where three clusters, namely, “In favour of eating insects”, “Against eating insects”, and “Picky towards eating insects”, were identified. With the same approach, the three clusters found in the present study could be named “In favour of eating jellyfish” (Cluster 1), “Against eating jellyfish” (Cluster 2), and “Picky towards eating jellyfish” (Cluster 3). The presence of Cluster 1 is encouraging since it indicates that at least one segment of consumers would accept jellyfish as novel food in the case of their future introduction in the market. Moreover, the results of Cluster 2 suggest that particular attention will be necessary to develop jellyfish products considering consumer preferences for different jellyfish body parts and presentation forms.

The effect of the anatomical part was also investigated in the United States through a sensory test carried out on laboratory-processed cannonball jellyfish. The results showed that inexperienced panellists did not discriminate among all samples tested in terms of overall preference, and, in particular, they accepted the leg tissue and the umbrella part of the cannonball jellyfish at the same level [31].

### 4.2. Sensory Perception and Drivers of Liking

Since scarce information on sensory descriptors for edible jellyfish is available in the literature, this work contributed to increasing knowledge in this regard. Conducting a preliminary focus group allowed for the development of a list of 40 sensory terms specific to jellyfish that were used in the consumer test. The adopted approach was different from the one previously reported [16], which collected a list of attributes related to jellyfish and seafood from the literature and selected the 20 most appropriate by applying a rate-all-that-apply test with a group of panellists. Even though different jellyfish species were evaluated (*Rhopilema esculentum* vs. *Rhizostoma Pulmo*) in the two studies, the different approaches to term selection identified nine common attributes including bitter, salty, sour, and umami for taste; fish, seaweed, and shellfish for flavour; and crunchy and hard for texture. Moreover, three attributes with similar meanings were observed between the two studies including marine/brackish flavour vs. sea flavour, gelatinous vs. jelly-like, and watery vs. juicy. Also comparing the vocabulary used in the present study with that of a work conducted in Portugal on pâté snack from *Catostylus tagi* umbrella [3], some common attributes (white colour, bitterness, saltiness, freshness, marine flavour, and shellfish flavour) and similar attributes (amber vs. cream-brownish colour and watery vs. juiciness) were observed. The number of attributes used in the present study (n = 40) was much higher than those in both of the above-mentioned studies, which were 20 [16] and 15 [3]. This large difference could be due to the type of sensory method that was applied to describe the sensory properties of jellyfish samples. Both of the last cited studies applied descriptive analysis, which is usually performed with fewer attributes than with the number of attributes that can be considered in a rapid profiling technique, such as the CATA test [24] used in the present work.

Since taste, smell, and texture were found to be both strong drivers of fish consumption and main barriers to the acceptance of fish [32], they could be expected to also play a great role in shaping consumer acceptance of jellyfish. However, the literature lacks information on this point for Western consumers. In the present study, the appearance of jellyfish influenced the hedonic response of the cluster named “Picky towards eating jellyfish” (Cluster 3), revealing that transparency acted as a driver of liking while an amber colour was a driver of disliking. This result is in accordance with the fact that colour is considered a quality index, varying from white to yellow to brown as jellyfish age, with the latter being unacceptable [9]. Regarding taste, umami was a significant driver of liking for all the subjects and Cluster 1. Saltiness tended to increase the liking of jellyfish (even if not significantly) for all the subjects and was a significant driver of liking for Cluster 2. These results are not surprising because it is well known that salty and umami are innately liked tastes by human subjects [33]. Moreover, the fact saltiness was significant for only one cluster was partially in line with a work on wild-caught fish that revealed a clear preference for a salty taste only by a segment of respondents [34]. However, our results are not in accordance with the same cross-cultural study reporting that Italian consumers prefer a neutral taste in fish. With respect to flavour, stagnant water was the attribute with the highest negative impact on liking. This term was previously used in some works on the sensory characterization of fish without any negative or positive connotation or relation with hedonic estimation [35,36]. It could be assumed that the stagnant water flavour was probably perceived as a sensory defect or undesired attribute and may be associated with a product that is not fresh. Thus, it is possible to suppose that consumers interpreted the attribute stagnant water attribute oppositely to the fresh/cool attribute, which instead showed a significant positive effect on liking. Regarding the texture, crunchiness was a relevant driver of liking. This result was in agreement with the traditional preference for the peculiar crunchy texture of jellyfish preserved in brine [9]. On the contrary, hardness significantly and negatively influenced liking. This is in contrast with the usual appreciation of the typical hard texture of traditional Chinese jellyfish products by Asian consumers [9,16]. Moreover, the watery attribute played a different role based on the cluster of consumers, being a driver of liking for Cluster 1 and a driver of disliking for Cluster 2, clearly indicating that consumers have distinguished preferences for jellyfish texture. These variable results of the effect of texture on the hedonic response are understandable given the large individual variability in texture preferences generally observed for food [37,38,39].

### 4.3. Emotions Associated with Jellyfish as Food

Consumers’ eating habits are influenced by their emotions, which can affect the foods they choose to consume and how much they eat [40]. Several positive emotions were linked to jellyfish consumption including adventurous, interested, satisfied, calm, and enthusiastic. This agreed with previous studies on novel foods showing that other alternative proteins were associated with positive emotions [41], in particular, with feelings of adventure and interest [19]. Positive emotions were suggested to be one of the most important factors influencing the decision to taste new foods [42] and the intention to eat alternative protein sources and help their increase acceptance [43]. In agreement with this, Cluster 1, which obtained the highest mean liking scores for all jellyfish samples, had a significantly higher proportion of positive emotions including enthusiastic, free, happy, pleasant, and satisfied. Contrarily, Cluster 2, which showed the lowest mean liking scores for jellyfish samples, was significantly more associated with negative emotions including disgusted and worried. The result that unpleasant feelings, such as disgust, were associated with novel and unfamiliar foods and could influence behaviour was supported by additional research [26].

Furthermore, the worried emotion was significantly more associated with the pieced samples, while positive emotions including free and glad were mainly associated with consuming the minced umbrella and minced arm samples, respectively. This result indicated that reducing the size of the jellyfish sample can limit negative emotions, probably because the level of visibility decreases. This result confirmed the important role played by the visible appearance of jellyfish previously observed in expectation conditions for Italian subjects [19] and Latin American consumers [20], who declared a high willingness to eat jellyfish in pieces and as a derivative product. Ultimately, the results supported the notion that Western consumers tend to increase their acceptance of novel foods when the level of visibility decreases [30,44].

### 4.4. Jellyfish Food Pairings

In this study, besides the sensory tests, jellyfish pairings were investigated. In recent years, there has been an increasing interest in food pairings within the scientific community [45]. In this light, the contribution of sensory science in the evaluation of food pairings has been highlighted [46]. Nevertheless, limited research has focused on novel food pairings, especially on alternative protein sources. Food products are seldom eaten alone; thus, understanding how consumers tend to pair a specific food with other ingredients, food products, and beverages is extremely helpful in food development and innovation. Even if this concept is generally valid for the food industry, service, and market, it has a more relevant meaning when novel or unfamiliar foods, such as jellyfish, are considered because of the disgust and neophobia frequently associated with these products [26]. Indeed, many food product developers and restaurant chefs find it difficult to identify and design ideal food pairings that are accepted by consumers [47]. An appropriate combination of food products and ingredients can improve the sensory acceptability of novel foods, contributing to positive experiences and consumer satisfaction [48]. Regarding jellyfish, some preliminary information on potential food pairings was collected in Italy [22] and Latin America [20], but this information referred to only expectation conditions since the studies were run online. In particular, the Italian research recommended verifying the obtained results with sensory tests since it was highlighted that consumers’ responses in expectation situations could be poor predictors of real food consumption contexts [49]. Thus, the present study is the first to investigate food pairings after a real tasting of jellyfish samples. Based on the results, among the most chosen food pairings (cooked vegetables, fish, pasta, potatoes, raw vegetables, rice/risotto, sauces/condiments, savoury snacks, and spices/aromatic herbs), vegetables, pasta, rice/risotto, and potatoes agreed with the jellyfish food pairing expectations previously expressed by Italian females [22]. On the contrary, Italians tended to avoid pairing jellyfish with beverages, fruits, dairy, and fatty products, as previously observed for the respondents from the southernmost Latin American countries (Uruguay, Argentina, and Chile) [20]. Additionally, the observed significant effect of the jellyfish samples on food pairings suggested that different combinations of jellyfish body parts and presentation forms should be taken into account in the future development of new food products to meet consumer expectations, which can vary depending on the gastronomic use of jellyfish.

## 5. Conclusions

This research provided, for the first time, scientific information concerning the perception of and affective response to jellyfish as a food source by Italian consumers. In particular, the results highlighted the effect of the presentation form of jellyfish on acceptance (with striped and minced samples more liked than pieced samples) while no effect of body part on the affective response was observed. Secondly, the study allowed us to identify the sensory attributes and the emotions driving the liking of jellyfish samples. Thirdly, the most promising food pairings for jellyfish were revealed. Moreover, three clusters with different preferences, sensory drivers of liking, and emotions associated with jellyfish were identified. Overall, this study provided new findings useful for many food researchers, developers, and innovators interested in the transition towards alternative protein sources, including innovative jellyfish-based products and dishes that fulfil consumers’ needs and expectations.

## Figures and Tables

**Table 1:** Characteristics of all subjects and the three identified clusters of subjects.

		Total (n = 106)	Cluster 1 (n = 40)	Cluster 2 (n = 44)	Cluster 3 (n = 22)	χ2	*p*
		%	%	%	%		
Gender						0.701	0.704
	Male	42.5	47.5	38.6	40.9		
	Female	57.5	52.5	61.4	59.1		
Age						2.311	0.315
	18–30	91.5	85.0	95.5	95.5		
	31–45	8.5	15.0	4.5	4.5		
Nationality						0.898	0.638
	Italian	78.3	82.5	77.3	72.7		
	Non-Italian	21.7	17.5	18.2	27.3		
Food habits						4.959	0.292
	Omnivore	64.2	60.0	70.5	59.1		
	Flexitarian	34.9	40.0	29.5	36.4		
	Vegetarian	0.9	0.0	0.0	4.5		
	Vegan	0.0	0.0	0.0	0.0		
Previous tasting of jellyfish						3.888	0.143
	Yes	16.0	25.0	11.4	9.1		
	No	84.0	75.0	88.6	90.9		

**Table 2:** Lists of the attributes, emotions and food pairings used during the check-all-that-applied tests.

Attributes	Emotions	Food Pairings
Amber	Active	Bakery products
Grainy	Adventurous	Cheese
Opaque/haze	Affectionate	Cooked vegetables
Smooth	Aggressive	Cream
Small dots	Angry	Dessert
Transparent	Bored	Eggs
Uniform colour	Calm	Fish
White	Daring	Fruits
Wrinkled/rough	Disgusted	Honey/jam
Bitter	Energetic	Meat
Salty	Enthusiastic	Pasta
Sour	Free	Potatoes
Sweet	Friendly	Pulses
Umami	Glad	Raw vegetables
Fish	Good	Rice/risotto
Fresh/cool	Good-natured	Sauces/condiments
Green vegetables	Guilty	Savoury snacks
Iron	Happy	Spices/aromatic herbs
Marine/brackish	Interested	Yogurt
Mineral	Joyful	None, I would eat it alone
Pet food	Loving	None, I would not eat it
Seaweed	Merry	
Shellfish	Mild	
Stagnant water	Nostalgic	
Sulfur	Peaceful	
Toasted	Pleasant	
Vinegar	Pleased	
Cartilaginous	Polite	
Compact/dense	Quiet	
Crunchy	Satisfied	
Dry	Secure	
Elastic	Steady	
Fibrous	Tame	
Gelatinous	Tender	
Hard	Understanding	
Porous	Warm	
Slimy	Whole	
Soft	Wild	
Viscous	Worried	
Watery		

**Table 3:** Consumers overall liking mean values obtained from all subjects and three clusters for the six samples of jellyfish differing in terms of parts (arms, umbrella) and presentation form (pieced, striped, minced).

Variable	Total (n = 106)	Cluster 1 (n = 40, 37.7%)	Cluster 2 (n = 44, 41.5%)	Cluster 3 (n = 22, 20.8%)
Part				
Umbrella	4.5 A	5.6 Aa	3.1 Bb	5.1 Aa
Arms	4.4 A	5.7 Aa	3.7 Ab	3.7 Bb
*p*-value	0.689	0.743	0.002	<0.0001
Presentation form				
Pieced	4.0 B	5.3 Ba	3.0 Bb	3.5 Bb
Striped	4.6 A	5.9 Aa	3.6 Ac	4.6 Ab
Minced	4.7 A	5.7 ABa	3.6 Ac	5.0 Ab
*p*-value	<0.0001	0.045	0.015	<0.0001
Part*Presentation form				
Pieced umbrella	4.0 B	5.0 Aa	2.8 Bb	4.6 Ba
Pieced arms	3.9 B	5.5 Aa	3.2 Bb	2.5 Cb
Striped umbrella	4.7 A	5.8 Aa	3.5 ABb	4.9 Ba
Striped arms	4.7 A	5.9 Aa	3.7 ABb	4.4 Bb
Minced umbrella	4.7 A	5.9 Aa	3.0 Bb	5.9 Aa
Minced arms	4.7 A	5.6 Aa	4.2 Ab	4.0 Bb
*p*-value	<0.0001	0.114	0.0002	<0.0001

Different capital letters within columns indicate significantly different mean values (Tukey’s HSD test, *p* < 0.05). Different lower letters within rows indicate significantly different mean values (Tukey’s HSD test, *p* < 0.0001).

**Table 4:** Sensory attributes selected for six jellyfish samples differing in terms of body parts (arms, umbrella) and presentation form (pieced, striped, minced) during the check-all-that-apply test. For each attribute, the Cochran’s Q test *p*-value, the occurrences (OC, percentage in brackets) and the significance values from the multiple pairwise comparison tests (Sheskin—S) are reported.

Attributes	*p*-Value	Pieced Umbrella			Pieced Arms			Striped Umbrella			Striped Arms			Minced Umbrella			Minced Arms		
		OC	S		OC	S		OC	S		OC	S		OC	S		OC	S	
Appearance																			
**Amber**	**<0.0001**	**3**	**0.028**	**(a)**	**42**	**0.396**	**(b)**	**2**	**0.019**	**(a)**	**41**	**0.387**	**(b)**	**1**	**0.009**	**(a)**	**45**	**0.425**	**(b)**
**Grainy**	**<0.0001**	**6**	**0.057**	**(a)**	**8**	**0.075**	**(a)**	**5**	**0.047**	**(a)**	**11**	**0.104**	**(a)**	**33**	**0.311**	**(b)**	**45**	**0.425**	**(b)**
**Opaque/haze**	**0.013**	**28**	**0.264**	**(ab)**	**32**	**0.302**	**(b)**	**22**	**0.208**	**(ab)**	**18**	**0.170**	**(ab)**	**26**	**0.245**	**(ab)**	**17**	**0.160**	**(a)**
**Smooth**	**0.001**	**17**	**0.160**	**(b)**	**10**	**0.094**	**(ab)**	**11**	**0.104**	**(ab)**	**13**	**0.123**	**(ab)**	**4**	**0.038**	**(a)**	**2**	**0.019**	**(a)**
**Small dots**	**<0.0001**	**14**	**0.132**	**(a)**	**2**	**0.019**	**(a)**	**11**	**0.104**	**(a)**	**5**	**0.047**	**(a)**	**39**	**0.368**	**(b)**	**6**	**0.057**	**(a)**
**Transparent**	**<0.0001**	**39**	**0.368**	**(bc)**	**15**	**0.142**	**(a)**	**56**	**0.528**	**(c)**	**35**	**0.330**	**(b)**	**39**	**0.368**	**(bc)**	**10**	**0.094**	**(a)**
**Uniform colour**	**<0.0001**	**28**	**0.264**	**(bc)**	**34**	**0.321**	**(c)**	**17**	**0.160**	**(ab)**	**21**	**0.198**	**(bc)**	**1**	**0.009**	**(a)**	**21**	**0.198**	**(bc)**
**White**	**<0.0001**	**31**	**0.292**	**(b)**	**3**	**0.028**	**(a)**	**25**	**0.236**	**(b)**	**3**	**0.028**	**(a)**	**29**	**0.274**	**(b)**	**0**	**0.000**	**(a)**
**Wrinkled/rough**	**0.001**	**5**	**0.047**	**(ab)**	**13**	**0.123**	**(b)**	**0**	**0.000**	**(a)**	**12**	**0.113**	**(b)**	**8**	**0.075**	**(ab)**	**8**	**0.075**	**(ab)**
Taste																			
Bitter	0.972	4	0.038	(a)	4	0.038	(a)	3	0.028	(a)	4	0.038	(a)	3	0.028	(a)	5	0.047	(a)
**Salty**	**<0.0001**	**76**	**0.717**	**(a)**	**91**	**0.858**	**(bc)**	**80**	**0.755**	**(ab)**	**90**	**0.849**	**(abc)**	**83**	**0.783**	**(ab)**	**98**	**0.925**	**(c)**
Sour	0.319	3	0.028	(a)	7	0.066	(a)	7	0.066	(a)	3	0.028	(a)	6	0.057	(a)	4	0.038	(a)
Sweet	0.205	5	0.047	(a)	2	0.019	(a)	7	0.066	(a)	4	0.038	(a)	4	0.038	(a)	1	0.009	(a)
**Umami**	**0.008**	**20**	**0.189**	**(a)**	**22**	**0.208**	**(a)**	**25**	**0.236**	**(a)**	**33**	**0.311**	**(a)**	**21**	**0.198**	**(a)**	**34**	**0.321**	**(a)**
Flavour																			
Fish	0.232	28	0.264	(a)	36	0.340	(a)	28	0.264	(a)	38	0.358	(a)	28	0.264	(a)	29	0.274	(a)
Fresh/cool	0.210	28	0.264	(a)	17	0.160	(a)	19	0.179	(a)	20	0.189	(a)	22	0.208	(a)	16	0.151	(a)
Green vegetables	0.438	6	0.057	(a)	7	0.066	(a)	3	0.028	(a)	7	0.066	(a)	3	0.028	(a)	4	0.038	(a)
**Iron**	**0.011**	**5**	**0.047**	**(a)**	**9**	**0.085**	**(a)**	**5**	**0.047**	**(a)**	**15**	**0.142**	**(a)**	**7**	**0.066**	**(a)**	**13**	**0.123**	**(a)**
Marine/brackish	0.197	49	0.462	(a)	53	0.500	(a)	42	0.396	(a)	57	0.538	(a)	51	0.481	(a)	51	0.481	(a)
Mineral	0.282	33	0.311	(a)	41	0.387	(a)	37	0.349	(a)	46	0.434	(a)	42	0.396	(a)	39	0.368	(a)
**Pet food**	**0.006**	**8**	**0.075**	**(a)**	**17**	**0.160**	**(a)**	**7**	**0.066**	**(a)**	**9**	**0.085**	**(a)**	**7**	**0.066**	**(a)**	**15**	**0.142**	**(a)**
Seaweed	0.664	42	0.396	(a)	47	0.443	(a)	40	0.377	(a)	48	0.453	(a)	42	0.396	(a)	47	0.443	(a)
**Shellfish**	**0.004**	**16**	**0.151**	**(a)**	**25**	**0.236**	**(ab)**	**18**	**0.170**	**(a)**	**34**	**0.321**	**(b)**	**28**	**0.264**	**(ab)**	**29**	**0.274**	**(ab)**
Stagnant water	0.518	25	0.236	(a)	25	0.236	(a)	19	0.179	(a)	24	0.226	(a)	30	0.283	(a)	25	0.236	(a)
Sulphur	0.160	5	0.047	(a)	4	0.038	(a)	3	0.028	(a)	11	0.104	(a)	6	0.057	(a)	6	0.057	(a)
Toasted	0.225	2	0.019	(a)	5	0.047	(a)	2	0.019	(a)	3	0.028	(a)	2	0.019	(a)	7	0.066	(a)
Vinegar	0.262	11	0.104	(a)	8	0.075	(a)	7	0.066	(a)	13	0.123	(a)	7	0.066	(a)	13	0.123	(a)
Texture																			
**Cartilaginous**	**<0.0001**	**78**	**0.736**	**(c)**	**65**	**0.613**	**(bc)**	**70**	**0.660**	**(bc)**	**60**	**0.566**	**(ab)**	**57**	**0.538**	**(ab)**	**48**	**0.453**	**(a)**
**Compact/dense**	**0.023**	**28**	**0.264**	**(ab)**	**30**	**0.283**	**(b)**	**22**	**0.208**	**(ab)**	**22**	**0.208**	**(ab)**	**20**	**0.189**	**(ab)**	**13**	**0.123**	**(a)**
**Crunchy**	**0.000**	**59**	**0.557**	**(bc)**	**48**	**0.453**	**(abc)**	**61**	**0.575**	**(c)**	**43**	**0.406**	**(ab)**	**59**	**0.557**	**(bc)**	**40**	**0.377**	**(a)**
Dry	0.487	5	0.047	(a)	5	0.047	(a)	4	0.038	(a)	2	0.019	(a)	4	0.038	(a)	1	0.009	(a)
**Elastic**	**0.006**	**19**	**0.179**	**(ab)**	**18**	**0.170**	**(ab)**	**31**	**0.292**	**(b)**	**20**	**0.189**	**(ab)**	**14**	**0.132**	**(a)**	**15**	**0.142**	**(a)**
Fibrous	0.097	21	0.198	(a)	20	0.189	(a)	19	0.179	(a)	13	0.123	(a)	20	0.189	(a)	11	0.104	(a)
**Gelatinous**	**0.010**	**28**	**0.264**	**(a)**	**44**	**0.415**	**(ab)**	**39**	**0.368**	**(ab)**	**41**	**0.387**	**(ab)**	**43**	**0.406**	**(ab)**	**49**	**0.462**	**(b)**
**Hard**	**<0.0001**	**41**	**0.387**	**(c)**	**29**	**0.274**	**(bc)**	**30**	**0.283**	**(bc)**	**19**	**0.179**	**(ab)**	**19**	**0.179**	**(ab)**	**7**	**0.066**	**(a)**
Porous	0.210	4	0.038	(a)	4	0.038	(a)	1	0.009	(a)	1	0.009	(a)	3	0.028	(a)	6	0.057	(a)
Slimy	0.244	15	0.142	(a)	21	0.198	(a)	17	0.160	(a)	26	0.245	(a)	18	0.170	(a)	18	0.170	(a)
Soft	0.676	4	0.038	(a)	7	0.066	(a)	3	0.028	(a)	7	0.066	(a)	4	0.038	(a)	6	0.057	(a)
**Viscous**	**0.025**	**9**	**0.085**	**(a)**	**22**	**0.208**	**(b)**	**15**	**0.142**	**(ab)**	**15**	**0.142**	**(ab)**	**9**	**0.085**	**(a)**	**13**	**0.123**	**(ab)**
**Watery**	**0.044**	**23**	**0.217**	**(a)**	**37**	**0.349**	**(a)**	**24**	**0.226**	**(a)**	**37**	**0.349**	**(a)**	**33**	**0.311**	**(a)**	**30**	**0.283**	**(a)**

The attributes identified as significant in Cochran’s Q test are reported in bold. In the rows, values identified by different lowercase superscript letters indicate significant differences identified with Sheskin’s multiple pairwise comparison tests.

**Table 5:** Emotions that arose when tasting the six jellyfish samples differing in terms of body parts (arms, umbrella) and presentation form (pieced, striped, minced) during the check-all-that-apply test. For each attribute, the Cochran’s Q test *p*-value, the occurrences (OC—percentage in brackets), and the significance values from the multiple pairwise comparison tests (Sheskin—S) are reported.

Emotions	*p*	Pieced Umbrella			Pieced Arms			Striped Umbrella			Striped Arms			Minced Umbrella			Minced Arms		
		OC	S		OC	S		OC	S		OC	S		OC	S		OC	S	
Active	0.480	10	0.094	(a)	8	0.075	(a)	8	0.075	(a)	15	0.142	(a)	11	0.104	(a)	13	0.123	(a)
Adventurous	0.661	35	0.330	(a)	41	0.387	(a)	35	0.330	(a)	33	0.311	(a)	37	0.349	(a)	33	0.311	(a)
Affectionate	0.649	1	0.009	(a)	2	0.019	(a)	0	0	(a)	1	0.009	(a)	1	0.009	(a)	1	0.009	(a)
Aggressive	0.549	7	0.066	(a)	4	0.038	(a)	7	0.066	(a)	4	0.038	(a)	9	0.085	(a)	8	0.075	(a)
Angry	0.133	4	0.038	(a)	1	0.009	(a)	5	0.047	(a)	3	0.028	(a)	1	0.009	(a)	1	0.009	(a)
Bored	0.053	10	0.094	(a)	1	0.009	(a)	7	0.066	(a)	5	0.047	(a)	9	0.085	(a)	5	0.047	(a)
Calm	0.108	11	0.104	(a)	11	0.104	(a)	20	0.189	(a)	21	0.198	(a)	13	0.123	(a)	13	0.123	(a)
Daring	0.162	7	0.066	(a)	9	0.085	(a)	5	0.047	(a)	11	0.104	(a)	4	0.038	(a)	9	0.085	(a)
Disgusted	0.160	30	0.283	(a)	39	0.368	(a)	25	0.236	(a)	28	0.264	(a)	26	0.245	(a)	29	0.274	(a)
Energetic	0.746	12	0.113	(a)	7	0.066	(a)	10	0.094	(a)	10	0.094	(a)	7	0.066	(a)	9	0.085	(a)
Enthusiastic	0.085	13	0.123	(a)	12	0.113	(a)	18	0.170	(a)	21	0.198	(a)	15	0.142	(a)	10	0.094	(a)
**Free**	**0.086**	**3**	**0.028**	**(a)**	**9**	**0.085**	**(ab)**	**8**	**0.075**	**(ab)**	**9**	**0.085**	**(ab)**	**13**	**0.123**	**(b)**	**6**	**0.057**	**(ab)**
Friendly	0.672	3	0.028	(a)	5	0.047	(a)	6	0.057	(a)	7	0.066	(a)	5	0.047	(a)	3	0.028	(a)
**Glad**	**0.044**	**1**	**0.009**	**(a)**	**7**	**0.066**	**(ab)**	**10**	**0.094**	**(ab)**	**8**	**0.075**	**(ab)**	**6**	**0.057**	**(ab)**	**11**	**0.104**	**(b)**
Good	0.920	12	0.113	(a)	14	0.132	(a)	15	0.142	(a)	12	0.113	(a)	13	0.123	(a)	16	0.151	(a)
Good-natured	0.740	5	0.047	(a)	4	0.038	(a)	8	0.075	(a)	5	0.047	(a)	4	0.038	(a)	6	0.057	(a)
Guilty	0.805	4	0.038	(a)	3	0.028	(a)	2	0.019	(a)	2	0.019	(a)	4	0.038	(a)	3	0.028	(a)
Happy	0.076	5	0.047	(a)	4	0.038	(a)	13	0.123	(a)	7	0.066	(a)	11	0.104	(a)	11	0.104	(a)
Interested	0.259	44	0.415	(a)	42	0.396	(a)	52	0.491	(a)	52	0.491	(a)	43	0.406	(a)	50	0.472	(a)
Joyful	0.647	4	0.038	(a)	3	0.028	(a)	8	0.075	(a)	5	0.047	(a)	5	0.047	(a)	6	0.057	(a)
Loving	0.580	1	0.009	(a)	2	0.019	(a)	4	0.038	(a)	3	0.028	(a)	3	0.028	(a)	1	0.009	(a)
Merry	0.063	1	0.009	(a)	0	0	(a)	4	0.038	(a)	0	0	(a)	1	0.009	(a)	1	0.009	(a)
Mild	0.455	6	0.057	(a)	2	0.019	(a)	4	0.038	(a)	4	0.038	(a)	7	0.066	(a)	7	0.066	(a)
Nostalgic	0.240	1	0.009	(a)	3	0.028	(a)	5	0.047	(a)	1	0.009	(a)	2	0.019	(a)	1	0.009	(a)
Peaceful	0.138	2	0.019	(a)	4	0.038	(a)	9	0.085	(a)	9	0.085	(a)	5	0.047	(a)	4	0.038	(a)
Pleasant	0.333	8	0.075	(a)	4	0.038	(a)	8	0.075	(a)	8	0.075	(a)	3	0.028	(a)	5	0.047	(a)
Pleased	0.263	7	0.066	(a)	5	0.047	(a)	12	0.113	(a)	12	0.113	(a)	10	0.094	(a)	12	0.113	(a)
Polite	0.144	3	0.028	(a)	3	0.028	(a)	4	0.038	(a)	8	0.075	(a)	1	0.009	(a)	5	0.047	(a)
Quiet	0.579	8	0.075	(a)	5	0.047	(a)	12	0.113	(a)	10	0.094	(a)	8	0.075	(a)	9	0.085	(a)
**Satisfied**	**0.027**	**8**	**0.075**	**(ab)**	**6**	**0.057**	**(a)**	**19**	**0.179**	**(b)**	**9**	**0.085**	**(ab)**	**13**	**0.123**	**(ab)**	**10**	**0.094**	**(ab)**
Secure	0.677	10	0.094	(a)	6	0.057	(a)	8	0.075	(a)	6	0.057	(a)	6	0.057	(a)	10	0.094	(a)
Steady	0.588	2	0.019	(a)	4	0.038	(a)	4	0.038	(a)	5	0.047	(a)	1	0.009	(a)	3	0.028	(a)
Tame	0.251	3	0.028	(a)	1	0.009	(a)	2	0.019	(a)	0	0	(a)	0	0	(a)	1	0.009	(a)
Tender	0.520	2	0.019	(a)	3	0.028	(a)	3	0.028	(a)	5	0.047	(a)	1	0.009	(a)	3	0.028	(a)
Understanding	0.614	12	0.113	(a)	10	0.094	(a)	12	0.113	(a)	15	0.142	(a)	13	0.123	(a)	16	0.151	(a)
Warm	0.649	4	0.038	(a)	3	0.028	(a)	2	0.019	(a)	6	0.057	(a)	4	0.038	(a)	3	0.028	(a)
Whole	0.267	3	0.028	(a)	1	0.009	(a)	1	0.009	(a)	0	0	(a)	1	0.009	(a)	0	0	(a)
Wild	0.308	14	0.132	(a)	22	0.208	(a)	16	0.151	(a)	24	0.226	(a)	21	0.198	(a)	18	0.170	(a)
**Worried**	**0.019**	**24**	**0.226**	**(a)**	**29**	**0.274**	**(a)**	**14**	**0.132**	**(a)**	**14**	**0.132**	**(a)**	**22**	**0.208**	**(a)**	**17**	**0.160**	**(a)**

The attributes identified as significant using Cochran’s Q test and the two-way ANOVA model are reported in bold. In the rows, values identified by different lowercase superscript letters indicate significant differences identified using Sheskin’s multiple pairwise comparison tests.

**Table 6:** Occurrences of the 39 emotions obtained from the three identified clusters of subjects for the six jellyfish samples in total (χ2 = 202.6; *p* = 0.0001).

Emotions	Cluster 1 (n = 40, 37.7%)		Cluster 2 (n = 44, 41.5%)		Cluster 3 (n = 22, 20.8%)	
Active	33		22		10	
Affectionate	1		1		4	**>**
Adventurous	72	**<**	96	**>**	46	
Aggressive	16		11		12	
Bored	17		16		4	
Calm	43		24		22	
Daring	23		12		10	
Disgusted	32	**<**	106	**>**	39	
Angry	6		5		4	
Energetic	30		11	**<**	14	
Enthusiastic	49	**>**	23	**<**	17	
Free	31	**>**	12		5	
Friendly	13		7		9	
Glad	25		13		5	
Good	44		25		13	
Good-natured	13		11		8	
Guilty	7		10		1	
Happy	33	**>**	10	**<**	8	
Interested	116		102		65	
Joyful	16		8		7	
Loving	8		5		1	
Merry	1		5		1	
Mild	13		8		9	
Nostalgic	9		3		1	
Peaceful	13		11		9	
Pleasant	37	**>**	12	**<**	9	
Pleased	18		9		9	
Polite	14		7		3	
Quiet	15	**<**	25		12	
Satisfied	39	**>**	13	**<**	13	
Secure	24		10	**<**	12	
Steady	7		7		5	
Tame	4		3		0	
Tender	9		5		3	
Understanding	37		25		16	
Warm	13		7		2	
Whole	5		1		0	
Wild	41		53	**>**	21	
Worried	39	**<**	61	**>**	20	

< and > indicate that the observed value is significantly lower or higher than the expected theoretical value, and mean values in bold in each row for each socio-demographic characteristic indicate statistically significant differences (χ2 per cell significant for α < 0.05; Fisher’s exact probability test, *p* < 0.05).

**Table 7:** Possible pairings identified when tasting jellyfish during the CATA test. For each attribute, the Cochran’s Q test *p*-value, the occurrence (OC), and the significance values from the multiple pairwise comparison tests (Sheskin—S) are reported.

Food Pairings	*p*	Pieced Umbrella			Pieced Arms			Striped Umbrella			Striped Arms			Minced Umbrella			Minced Arms			
		OC	S		OC	S		OC	S		OC	S		OC	S		OC	S		Total OC
Bakery products	0.219	2	0.019	(a)	5	0.047	(a)	5	0.047	(a)	5	0.047	(a)	8	0.075	(a)	2	0.019	(a)	27
Cheese	0.731	6	0.057	(a)	5	0.047	(a)	7	0.066	(a)	10	0.094	(a)	7	0.066	(a)	9	0.085	(a)	44
Cooked vegetables	0.437	23	0.217	(a)	26	0.245	(a)	29	0.274	(a)	31	0.292	(a)	23	0.217	(a)	29	0.274	(a)	161
Cream	0.833	2	0.019	(a)	5	0.047	(a)	3	0.028	(a)	4	0.038	(a)	4	0.038	(a)	5	0.047	(a)	23
Dessert	0.886	2	0.019	(a)	2	0.019	(a)	3	0.028	(a)	1	0.009	(a)	3	0.028	(a)	3	0.028	(a)	14
Eggs	0.282	7	0.066	(a)	7	0.066	(a)	9	0.085	(a)	9	0.085	(a)	14	0.132	(a)	13	0.123	(a)	59
Fish	0.078	19	0.179	(a)	21	0.198	(a)	31	0.292	(a)	31	0.292	(a)	25	0.236	(a)	29	0.274	(a)	156
Fruits	0.758	5	0.047	(a)	7	0.066	(a)	4	0.038	(a)	4	0.038	(a)	4	0.038	(a)	4	0.038	(a)	28
Honey/jam	0.380	9	0.085	(a)	8	0.075	(a)	10	0.094	(a)	7	0.066	(a)	7	0.066	(a)	3	0.028	(a)	44
Meat	0.688	4	0.038	(a)	4	0.038	(a)	6	0.057	(a)	8	0.075	(a)	4	0.038	(a)	5	0.047	(a)	31
**Pasta**	**0.000**	**11**	**0.104**	**(a)**	**11**	**0.104**	**(a)**	**23**	**0.217**	**(ab)**	**17**	**0.160**	**(ab)**	**19**	**0.179**	**(ab)**	**29**	**0.274**	**(b)**	**110**
**Potatoes**	**0.006**	**10**	**0.094**	**(a)**	**14**	**0.132**	**(ab)**	**21**	**0.198**	**(ab)**	**20**	**0.189**	**(ab)**	**20**	**0.189**	**(ab)**	**25**	**0.236**	**(b)**	**110**
**Pulses**	**0.038**	**1**	**0.009**	**(a)**	**4**	**0.038**	**(a)**	**8**	**0.075**	**(a)**	**6**	**0.057**	**(a)**	**8**	**0.075**	**(a)**	**3**	**0.028**	**(a)**	**30**
Raw vegetables	0.624	24	0.226	(a)	23	0.217	(a)	26	0.245	(a)	27	0.255	(a)	20	0.189	(a)	28	0.264	(a)	148
**Rice/risotto**	**0.001**	**18**	**0.170**	**(a)**	**24**	**0.226**	**(ab)**	**27**	**0.255**	**(ab)**	**32**	**0.302**	**(ab)**	**38**	**0.358**	**(b)**	**38**	**0.358**	**(b)**	**177**
Sauces/condiments	0.129	19	0.179	(a)	19	0.179	(a)	25	0.236	(a)	22	0.208	(a)	32	0.302	(a)	24	0.226	(a)	141
Savoury snacks	0.264	11	0.104	(a)	13	0.123	(a)	18	0.170	(a)	15	0.142	(a)	18	0.170	(a)	21	0.198	(a)	96
Spices/aromatic herbs	0.049	20	0.189	(a)	22	0.208	(a)	34	0.321	(a)	23	0.217	(a)	24	0.226	(a)	21	0.198	(a)	144
Yogurt	0.569	3	0.028	(a)	1	0.009	(a)	3	0.028	(a)	5	0.047	(a)	3	0.028	(a)	5	0.047	(a)	20
None, I would eat it alone	0.587	7	0.066	(a)	5	0.047	(a)	10	0.094	(a)	8	0.075	(a)	8	0.075	(a)	5	0.047	(a)	43
**None, I would not eat it**	**<0.0001**	**41**	**0.387**	**(b)**	**41**	**0.387**	**(b)**	**19**	**0.179**	**(a)**	**25**	**0.236**	**(ab)**	**27**	**0.255**	**(ab)**	**18**	**0.170**	**(a)**	**171**

The attributes identified as significant using the Cochran’s Q test are reported in bold. In the rows, values identified by different lowercase superscript letters indicate significant differences identified using Sheskin’s multiple pairwise comparison tests.

## Data Availability

The original contributions presented in the study are included in the article, further inquiries can be directed to the corresponding author.

## Supplementary Materials

The following supporting information can be downloaded at: https://www.mdpi.com/article/10.3390/foods13121872/s1, Material S1: Informed consent.
