# Supplementary material for: Effect of Jellyfish Body Parts and Presentation Form on Consumers Liking, Sensory Perception, Emotions, and Food Pairings
Source: Foods. 2024 Jun 14;13(12):1872. doi: 10.3390/foods13121872 (PMC11203045; doi:10.3390/foods13121872)
Supplement: Supplementary file 1 [file foods-13-01872-s001.zip › foods-3045018-supplementary.pdf]

Titolo studio: **Risposta affettiva e attitudine dei consumatori nei confronti del potenziale utilizzo gastronomico di diverse parti di medusa**

Io sottoscritto/a *(nome e cognome)* ______________________________

nato il *(data di nascita)* ______________________________

nato a *(luogo di nascita)* ______________________________

Dichiaro di:

- partecipare volontariamente allo studio di cui mi sono stati spiegati e di cui ho compreso lo scopo, le procedure alle quali potrò essere esposto, i possibili rischi e i benefici;

Pertanto autorizzo:

- ai sensi del Decreto legislativo 30.6.03 n. 196 codice privacy, il proponente di questo studio, a sottoporre i dati personali forniti allo sperimentatore, in quanto necessari alla mia partecipazione allo studio in oggetto, ai sensi del Decreto legislativo 30.6.03 n. 196 codice privacy;
- l'utilizzo dei dati relativi alla mia persona, in forma anonima e aggregata, in pubblicazioni scientifiche ed eventi divulgativi.

Pollenzo, (data) ____________________ Firma

____

Io sottoscritta LUISA TORRI dichiaro:

- che il soggetto ha firmato spontaneamente la sua partecipazione allo studio;
- di aver fornito al soggetto esaurienti spiegazioni in merito alle finalità dello studio, alle procedure, ai possibili rischi e benefici;
- di aver consegnato al soggetto stesso una copia del presente modulo, firmato e datato.

Pollenzo, (data) ____________________ Firma

____

UNISG - Università degli Studi di Scienze Gastronomiche
Piazza Vittorio Emanuele, 9 - Pollenzo - 12042 Bra (CN) - Italia
Telefono: +39 0172 458511 - Fax: +39 0172 458500
[illegible]
